# A Guide for Pain Management in Low and Middle Income Communities. Managing the Risk of Opioid Abuse in Patients with Cancer Pain

**DOI:** 10.3389/fphar.2016.00042

**Published:** 2016-03-01

**Authors:** Joseph V. Pergolizzi, Gianpietro Zampogna, Robert Taylor, Edmundo Gonima, Jose Posada, Robert B. Raffa

**Affiliations:** ^1^Department of Medicine, Johns Hopkins University School of MedicineBaltimore, MD, USA; ^2^Department of Anesthesiology, Georgetown University School of MedicineWashington, DC, USA; ^3^Department of Pharmacology, Temple University School of MedicinePhiladelphia, PA, USA; ^4^NEMA ResearchNaples, FL, USA; ^5^Anesthesiologist, Pain and Palliative Care, Pain Specialist in Hospital MilitarBogota, Colombia; ^6^Psychiatry, Colombian National Board of NarcoticsBogota, Colombia; ^7^Department of Pharmaceutical Sciences, Temple University School of PharmacyPhiladelphia, PA, USA

**Keywords:** cancer pain, malignant pain, opioid analgesia, opioids, undertreatment of cancer pain, assessment of cancer pain

## Abstract

Most patients who present with cancer have advanced disease and often suffer moderate to severe pain. Opioid therapy can be safe and effective for use in cancer patients with pain, but there are rightful concerns about inappropriate opioid use even in the cancer population. Since cancer patients live longer than ever before in history (and survivors may have long exposure times to opioid therapy), opioid misuse among cancer patients is an important topic worthy of deeper investigation. Cancer patients with pain must be evaluated for risk factors for potential opioid misuse and aberrant drug-taking behaviors assessed. A variety of validated screening tools should be used. Of particular importance is the fact that pain in cancer patients changes frequently, whether it is related to their underlying disease (progression or remission), pain related to treatment (such as painful chemotherapy-induced peripheral neuropathy), and concomitant pain unrelated to cancer (such as osteoarthritis, headache, or back pain). Fortunately, clinicians can use universal precautions to help reduce the risk of opioid misuse while still assuring that cancer patients get the pain therapy they need. Another important new “tool” in this regard is the emergence of abuse-deterrent opioid formulations.

## Introduction

The Cali Cancer Registry (CCR) is the best-known population-based source for descriptive epidemiology of cancer in Colombia specifically and in South America at large; dating back to 1962, it is the oldest cancer registry in Latin America (1989). The main drawback to the CCR is that it offers data for the urbanized area of Cali, but does not provide a full picture of the large and diverse Colombian nation. A recent study of Colombian cancer incidence reported that the annual total numbers of all cases of cancer in Colombia (except for skin cancer) was 17,819 and 18,772 for men and women, respectively (Pineros et al., 2006). The most frequent cancers in Colombian men were prostate (45.8 per 100,000), stomach (36.0 per 100,000), and lung (20.0 per 100,000); for women, the most common cancers were the cervix uteri (36.8 per 100,000), breast (30.0 per 100,000), and stomach (20.7 per 100,000; Pineros et al., 2006). Most Colombians who present with cancer are in an advanced disease state at the time of diagnosis. This pattern of a high cancer prevalence and advanced disease at diagnosis is not uncommon in developing nations.

Pain is a common symptom of cancer, typically worsening with disease progression. Cancer survivors may likewise suffer from chronic pain syndromes associated with the cancer treatments, such as chemotherapy-induced neuropathic pain (Westerling, 2014). More than half of patients with metastatic or terminal cancer (64%) experience pain, about 59% of patients undergoing anticancer treatment experience pain, and about a third (33%) of cancer survivors report pain (Ripamonti et al., 2012). About 15% of ambulatory cancer patients and 70% of palliative patients have pain symptoms (Yennurajalingam et al., 2012). As more and more patients survive cancer for longer periods of time, pain treatments must evolve to meet these new needs. Pain in cancer patients can be particularly challenging to manage; it is often multimechanistic, severe or even very severe, and variable with disease progression and treatments. Moreoever, cancer patients often experience pain of multiple types at multiple locations.

Assessing and managing pain in advanced cancer patients falls under the broad therapeutic approach of palliative care. Physicians should identify and assess the pain, the underlying causes and the ongoing treatments, and individualize pain therapy for each patient, bearing in mind that the pain may change as the disease and/or treatment progress (Portenoy, 2011). An important pharmacotherapeutic modality for cancer pain remains opioid therapy.

## Opioid therapy

Due to concerns about opioid misuse and diversion, opioid therapy may be under-prescribed and cancer pain go under-treated. For example, based on opioid analgesic consumption, Colombia is using far less than what might be considered an adequate level of opioids for analgesia (actual use of 6.78 mEq in mg per capita in 2010 vs. 204.13 mEq “adequate” use; Duthey and Scholten, 2014). Indeed, opioid consumption patterns indicate that about two-thirds of the world's population (66%) has virtually no access to opioid analgesics and just 7.5% of the world's population has adequate consumption levels to meet its pain burden (Duthey and Scholten, 2014). In general, opioids are not prescribed as often as they ought to be in many developing nations.

There are many reasons for the uneven consumption patterns of opioid analgesics. Opioids are not widely available in some regions for legal, social, public health, economic, or cultural reasons. There is also reluctance to prescribe available opioid analgesics due to lack of physician training, concerns about addiction, regulatory restrictions, potential legal liabilities, and patient attitudes. The reticence to make use of available opioid analgesics has been termed “opiophobia”(Rhodin, 2006).

The appropriate care of cancer patients requires pain control; indeed, pain management has been stated to be a fundamental human right (Daher, 2010). In 1988, the World Health Organization (WHO) advocated the use of oral morphine for the management of moderate to severe cancer pain (World Health Organization, 1988). Balancing concerns about opioid misuse against the need for appropriate pain control has resulted in what has been called “opioid risk management.” This approach recognizes the inherent risks in opioid therapy, but weighs them against the needs of patients dealing with moderate to severe or very severe cancer pain. The goal of opioid risk management is to minimize the harms associated with opioid therapy while maintaining access to opioid therapy for the control of pain.

## Inappropriate use, misuse, and abuse of opioids

It has been estimated that over 10% of chronic pain patients misuse opioid analgesics, i.e., that they take the drugs inappropriately. Opioid misuse encompasses taking the drugs in any way not prescribed, whether intentional or inadvertent and whether or not harm results (Garland et al., 2013). Inappropriate use of opioids may include misuse (e.g., taking more drug than prescribed for a “bad day”), abuse (taking the drugs recreationally), diversion (selling the drugs rather than consuming them), and addiction. The fact is that most cancer patients who are prescribed opioid analgesics appropriately will not misuse them. However, some will misuse opioids and the identification of patients at risk for opioid misuse remains a challenge. This problem is exacerbated by the fact that risk factors change with time and situation (Pergolizzi et al., 2012).

The risk of opioid abuse specifically in cancer patients is a subject of ongoing study. As cancer treatment improves, patients live longer, many in chronic pain. Their exposure to opioids will increase and the risks of misuse may be expected to rise (Starr et al., 2010). It had long been accepted among clinicians that cancer patients were at relatively low risk for opioid misuse (Porter and Jick, 1980). There were many sound reasons for this thinking. Decades ago, cancer mortality was much higher and thus patients used opioids only for a short period of palliative care. The idea of a cancer patient surviving for years—and taking opioid pain relievers for years—is more common today. Although cancer patients are typically older (Starr et al., 2010), and older age mitigates the risk for inappropriate opioid use, adequately defining and describing opioid misuse, abuse, and addiction can be very difficult in a medically ill population (Starr et al., 2010).

## Risk factors for opioid abuse in cancer patients

Cancer patients typically have a heavy symptom burden and a high degree of disease-related stress. Cancer can put enormous strain on the patient and caregivers, incorporating anxiety about everything from morbidity and mortality to financial worries and fears about how the disease will impact the family structure. These factors can force patients to rely on coping mechanisms over the course of their illness, which may last many months or years. “Chemical coping” is a term used to describe a continuum of inappropriate opioid behaviors to help manage stress or other similar symptoms; chemical coping may range from mild forms of misuse with no harmful consequences to outright addictive behaviors (Del Fabbro, 2014). Although there are no studies about chemical coping in cancer patients of which the authors are aware, this would seem to be a particularly important area for future investigation.

In general, risk factors for inappropriate use of opioids appear to be the same for cancer and noncancer pain patients. These include a history of substance abuse, active substance abuse (including but not limited to alcoholism and/or illicit drug use), tobacco use, mental health disorders (such as depression and anxiety disorders), and psychological traumas (such as childhood abuse; Passik and Kirsh, 2004; Compton and Volkow, 2006; Sullivan et al., 2006; Hojsted and Sjogren, 2007; Jamison et al., 2011; Pergolizzi et al., 2012). Cancer patients are not immune to these risk factors. In fact, they may have higher rates of some of the risk factors (such as smoking) than the general population. Alcoholism is also thought to be more prevalent among cancer patients, although it is not always diagnosed (Bruera et al., 1995; Martin et al., 2002; Del Fabbro, 2014).

The conundrum with cancer patients is that even patients with clear risk factors for opioid misuse (such as an active addiction) may legitimately have moderate to severe pain associated with their malignancy. For terminally ill patients with severe pain, addictive disorders were once considered non-issues; the patient near death was given medications to stop the pain without concern about potential addiction. With patients surviving cancer for longer and longer periods of time, these issues are no longer minor. Moreover, unchecked substance abuse may exacerbate suffering in the palliative patient, complicate the management of other cancer-related symptoms, and have a distressing effect on the patient's family (Passik and Theobald, 2000). Thus, oncologists today may need to consult with pain specialists and experts in addiction to help manage pain in cancer patients at high risk for opioid misuse.

The four A's of pain management outcomes are: **a**nalgesia, **a**ctivities of daily living, **a**dverse events, and **a**berrant drug-taking behaviors (Passik and Weinreb, 2000). Physicians must assess pain, screen for risk, and then consider treatment options, including nonopioid treatments and nonpharmacological treatments. Owing to the nature of disease progression and cancer therapy, pain assessments must be performed regularly to adequately capture changing pain symptoms. Gourlay described universal precautions for prescribing opioid analgesics, including assessment of risk, informed consent, treatment agreements (spelling out in writing the expectations of the physician and clinic), and regular re-assessments of both pain and aberrant-drug taking behaviors (Gourlay et al., 2005).

Pain intensity must be evaluated frequently in cancer patients. Cancer patients often have multiple pain sites and each site should be assessed individually, noting that not all pain in cancer patients is necessarily related to the cancer. They may have osteoarthritis pain, for example. The duration of pain, intensity, and aggravating and/or mitigating factors should be noted. The patient should be encouraged to describe the pain at each site, prompted if necessary by the clinical team with such words as burning, dull, throbbing, electrical, “pins and needles,” stabbing, and so on. A full medical history should be obtained, including the other drugs as well as over-the-counter products or supplements the patient is taking.

To assess for the potential of opioid misuse, the physician must review specific topics with the patient known to be risk factors for opioid misuse and abuse (see Table 1). Of course, certain patients may not be forthcoming about their use of drugs and alcohol. In one survey, 27% of potential patients concealed their use of illicit drugs from a physician evaluating them for pain therapy (Manchikanti et al., 2004). Clinicians should be thorough, careful, and respectful to patients in going over these points, but be wary that many patients will be less than truthful (Gourlay et al., 2005).

**Table 1:** **A short overview of risk factors for opioid inappropriate use (Pergolizzi et al., 2012)**.

**Factor**	**Considerations**	**Comments**
Alcohol use	Present use, inappropriate use, and alcoholism are risk factors as is recent history of alcohol abuse (Ives et al., 2006).	CAGE assessment is a simple and useful tool (Parsons et al., 2008).
Cocaine use	Present or recent history of this drug is a strong predictor of opioid inappropriate use (Ives et al., 2006).	A history of substance abuse *more than five years in the past* is not associated with opioid inappropriate use (Passik et al., 2011).
Substance abuse (other)	Includes marijuana, stimulants, legal, and illicit drugs (Ives et al., 2006).	Benzodiazepines in particular are associated with opioid inappropriate use (Skurtveit et al., 2010). Thus, among patients with a history of drug abuse, risk can be stratified by type of drug (cocaine and benzodiazepines are higher risk than marijuana, for example).
Family history of substance abuse	May be a risk factor even if the patient does not abuse substances himself (Prasant et al., 2006).	Applies to first-degree relatives only.
Current prescription for opioids	Long-term use of opioids is a risk factor for inappropriate use (Edlund et al., 2007).	
Mental health and mood disorders	Includes dissociative disorders, bipolar disorders, depression, schizophrenia, post-traumatic stress disorders, and others (Pergolizzi et al., 2012).	Mental health disorders are prevalent among those with chronic pain syndromes (Manchikanti et al., 2002).
Gambling addiction	Gambling addiction has been associated as a risk for opioid inappropriate use (Petry et al., 2005).	
Legal problems	In particular, history of drunk driving, drug convictions, or motor vehicle collisions (Turk et al., 2008).	
Trauma	Being a victim of a crime or abuse (Khoury et al., 2010; Vaughn et al., 2010).	Sexual abuse in childhood is a risk for both sexes but the risk is higher for women (Kendler et al., 2000).
Young age	Younger individuals are at greater risk for opioid inappropriate use than older people (Manchikanti et al., 2006).	Age at first exposure to opioids may be a risk factor (risk is greater for younger exposure; Becker et al., 2008) and should be considered in addition to the patient's current age.

Documentation is a crucial part of pain therapy for any patient, including diagnostic results, pain assessment evaluations, patient history, risk assessments, treatment objectives, and discussions between the clinical team and patient about the risks and benefits of opioid therapy. Over the course of treatment, changes in the patient's condition, therapy, and evaluations should be assessed and carefully documented.

Aberrant drug behaviors are exhibited by patients prescribed opioids include (but are not limited to) demands for stronger or more analgesics, “lost” prescriptions (especially if this occurs frequently), demands to see the physician without an appointment, and insisting on specific pain relievers by product name and dosage (Passik and Kirsh, 2004). Aberrant drug-related behaviors can be deceptive. In other words, while these behaviors can be highly suggestive of misuse, some patients will honestly lose prescriptions or show up at the clinic without an appointment. Furthermore, in cancer patients, it is important to differentiate between addiction and pseudoaddiction, both of which may provoke aberrant drug-related behaviors. Pseudoaddiction occurs when the patient has inadequate analgesia and seeks greater pain relief; addiction occurs when the patient uses the drug compulsively, may have strong drug cravings, and has little control over drug use even when understanding that the drug is harmful (Katz et al., 2007). Both addiction and pseudoaddiction will result in patients complaining, sometimes quite assertively, that they need more and stronger pain relievers. Patients who are addicted to opioid pain relievers should be tapered off them; patients who are “pseudoaddicted” should be titrated and may actually need larger doses or different opioid analgesics.

Some of the outward signs suggestive of opioid misuse or abuse appear in Table 2. Note that these signs may occur in patients taking opioids as prescribed or may be attributable to other factors. Cancer patients, in particular, may exhibit signs related to their underlying disease or adverse effects of aggressive anticancer treatments that can mimic signs of abuse. Furthermore, cancer is a highly stressful condition that may cause patients to appear anxious, fearful, and agitated.

**Table 2:** **Signs that may suggest opioid inappropriate use and alternate explanations in cancer patients (Gourlay et al., 2005)**.

**Sign**	**Alternate explanation(s)**
Unkempt appearance	Patient may be depressed or overwhelmed by illness; patient may have symptoms related to cancer treatments that cause them to feel sick all of the time and thus neglect their appearance
Anxiety, nervousness, agitation	Patient may be upset about cancer, his or her prognosis, or have fears relating to treatment, home or hospital care, or financial worries
Sniffles	Allergies, cold, sinus problems, symptoms associated with cancer treatments
Watery eyes	Allergies, cold; symptoms associated with stress or fear
Cough	Cancer-related cough; allergies, cold, heart burn
Lethargy	Fatigue, depression, disease progression, lethargy related to treatments, especially chemotherapy, lethargy associated with other drugs the patient might be taking
Drowsiness, nodding off	Fatigue, disease progression, adverse effect related to treatment especially chemotherapy or other drug therapy
Skin lesions (from injecting drug)	Possibly related to other drug therapies associated with the cancer; skin disorders, rash

So-called aberrant drug-taking behaviors can also be suggestive of opioid abuse (Passik and Kirsh, 2004). These include patients with erratic habits (failing to show up for an appointment or coming to the clinic without an appointment), requesting appointments at the end of the day when they anticipate clinicians will be in a hurry, reluctance or refusal to undergo physical examination, failing to keep referral appointments, refusing to divulge past medical history, or unusual behaviors such as obvious agitation and emotional outbursts. Physicians should train the entire clinical team to be alert to such aberrant behaviors, but their “diagnostic power” is limited. While such behaviors suggest potential opioid misuse, they do not prove it. Not every cancer patient who is in a hurry or who has poor social skills is a drug abuser.

## Screening tools

Several assessment instruments have been developed to help clinicians better identify patients at risk of opioid misuse. These tools were not all developed specifically for cancer patients, but they can be used in this population.

### CAGE and CAGE-AID

The original CAGE survey dating back to 1974 has four questions related to alcohol use (Mayfield et al., 1974). It was **a**dapted to **i**nclude **d**rugs (AID) in the composite CAGE-AID instrument. Participants are asked to respond yes or no to four questions that give the survey its acronym: (1) are they trying to use less (“**c**utting down”), (2) are they annoyed that people criticize their substance use (“**a**nnoyance”), (3) do they feel guilty about their substance use (“**g**uilty”), and (4) do they need to use the substance first thing in the morning to get going (“**e**ye-opener”). The test can be used for alcohol alone, as originally intended, or for other substances (CAGE-AID). The rule of thumb is that one yes warrants further evaluation of the patient, and two or more yes answers strongly suggests substance abuse. The CAGE survey is easy to use and is familiar to most clinicians, but it may be of limited utility and accuracy in patients with psychiatric disorders (O'Connell et al., 2004).

### Addiction behaviors checklist

The Addiction Behaviors Checklist (ABC) was designed to evaluate patients prescribed opioid analgesics to manage persistent pain (Wu et al., 2006). The checklist is filled out by the clinician, who evaluates the patient at the current office visit and also notes relevant behavioral changes since the last visit. It is a succinct, targeted survey that takes about 10 min to complete. A score of 3 or more on the 20-question checklist suggests that opioids are being used inappropriately. A study of 136 chronic pain patients found it to be a viable assessment tool (Wu et al., 2006).

### Opioid risk tool

The Opioid Risk Tool (ORT) evaluates 10 psychosocial risk factors for opioid abuse, including history of substance abuse, sexual abuse, and mental health disorders. ORT scores classify patients as being at low, moderate, or high risk for opioid misuse. Notably, ORT was specifically designed and validated for use in cancer patients (Barclay et al., 2014).

### Screener and opioid assessment for patients with pain—short form

The Screener and Opioid Assessment for Patients with Pain—Short Form (SOAPP-SF), a subset the longer form of the original SOAPP 14-item questionnaire, is a 5-item questionnaire to be filled out by the patient. Patients who score 4 or more are considered to be at high risk for opioid abuse. SOAPP-SF has a sensitivity of 0.86 and specificity of 0.67 with a 33% chance of a false positive (Koyyalagunta et al., 2013).

### Pain assessment and documentation tool

The Pain Assessment and Documentation Tool (PADT) is a short survey completed by the patient which evaluates analgesia, activities of daily living, and adverse events. A checklist on the form is to be completed by the physician evaluating the patient's potential aberrant drug-related behaviors (including mood changes, reports of stolen prescriptions, arrests, medication hoarding, and so on).

## Understanding risks inherent in the cancer population

Ongoing or a recent history of substance abuse and certain mental health disorders are known risk factors for opioid misuse and abuse, but the specific prevalence of these conditions in the cancer population has not been thoroughly elucidated. Indeed, the cancer population has changed considerably in recent decades with more and more patients undergoing longer treatments and surviving longer with the disease, in remission, or cured. It has been estimated that about 20% to 50% of cancer patients have some psychiatric disorder, most frequently depression (Grassi et al., 1996, 2005; Pirl, 2004). Mental health disorders in cancer patients may relate to the patient's emotional response to a devastating diagnosis or it may be associated with the patient's general physical condition, possibly due to metabolic changes. About 10% to 30% of cancer patients are reported to have anxiety disorders (Mehnert et al., 2007; Brown et al., 2009), which often relates to their diagnosis and uncertainty about the future.

The use and abuse of alcohol has been associated with certain cancers, in particular, breast, colon, rectal, esophageal, larynx, liver, mouth, and pharynx cancer (Montoya, 2013). A study from Brazil found alcohol abuse rates of 17.2% and tobacco abuse rates of 27.3% for cancer patients (Polidoro Lima and Osorio, 2014). The rate can be much higher for specific cancers. For instance, epidemiological studies found that 70% of mouth cancer patients were heavy drinkers. Alcohol drinkers are more likely to smoke (Jegu et al., 2013) and smoking has well-known associations with cancer. Thus, certain cancer patients may have higher rates of certain risk factors for opioid abuse than do the general population.

Another concern in stratifying risk for opioid abuse in cancer patients is terminology related to longer-living cancer patients (Bell and Ristovski-Slijepcevic, 2013). For example, clinicians may use the term “cancer survivors” to describe either: those in treatment; those in periods of remission but expected to succumb to the disease; and those who may be cured (Harrop et al., 2011). Yet each category of survivor has different needs and burdens, including different risk levels for opioid misuse. Furthermore, many “cancer survivors” have chronic pain syndromes, that necessitate long-term opioid therapy. New definitions and methodologies must be developed to better evaluate risk factors for opioid abuse in diverse populations—for example, newly diagnosed cancer patients vs. patients currently in remission but dealing with chronic pain or treatment-related pain syndromes. Indeed, the subject of chronic pain in cancer survivors will be of increasing importance for pain specialists, as this population of patients is likely to increase in number and to have special needs with respect to long-term analgesia.

## Clinical monitoring

### Urine drug testing

Cancer patients on opioid therapy require regular clinical monitoring and assessment to maximize appropriate treatment and minimize misuse. Urine drug testing (UDT) is a simple, inexpensive, and familiar way to screen patients for inappropriate drug use (Pergolizzi et al., 2010). Clinicians must be aware of the specificity and sensitivity of the test methods; some tests, for example, may not detect synthetic opioids (Heit and Gourlay, 2004). Not all commercial UDT facilities screen for all substances; this must be discussed in advance with the laboratory. UDT for cancer patients prescribed an opioid is conducted in order to evaluate the metabolism of opioids (unlike other UDT, the presence of opioids in the urine in this case is appropriate) and the absence of illicit drugs. UDT should be discussed in advance with the patient. It may be done randomly, at clinician request, or on a set schedule. In addition to providing insight into the patient's use of opioids, it provides objective documentation of the patient's compliance with the treatment plan and can serve as an important conversation point with the patient. Physicians should resist the urge to draw definitive conclusions from one unexpected UDT result, but rather should use this as an opportunity to discuss drug therapy with the patient. False positives and false negatives are not uncommon.

In some instances, UDT results will not detect the prescribed opioid medication. There may be several reasons for this, including: the patient may be misusing their medications, either by binging (and thus not having any more left); the patient may be diverting the drugs and selling them rather than taking them; or, the patient may be noncompliant and not taking the drugs on “good days.” Any of these situations must be addressed. However, unexpected UDT results can also occur due to laboratory errors or screens that are not sensitive enough to detect the particular opioid in question. Unexpected or suspicious UDT results should be viewed first-and-foremost as an opportunity to discuss opioid-taking behaviors with the patient.

### Treatment agreements

A treatment agreement is a written document that spells out in plain language the goals, risks, and benefits of opioid therapy for a given patient. These agreements may be of great or limited utility, depending on how they are presented and administered (Anderson and Burris, 2010; Starrels et al., 2010). The main purpose of a treatment agreement is to make sure that the patient is fully aware of the risks as well as the benefits of opioid therapy and to specify how treatment will be conducted. It also serves as excellent documentation for the clinic that these matters were discussed frankly and clearly with the patient. For example, the treatment agreement should mention that UDT will be conducted and under what conditions (such as random UDT is part of therapy). It may also specify that patients can be subjected to periodic pill counts. The treatment agreement should also state what constitutes inappropriate opioid use and that there may be consequences to opioid misuse. The treatment agreement should also explain the risks of opioid therapy, including the potential side effects.

### Assessments

Cancer can be a progressive disease and involve aggressive treatment modalities. Therefore, pain in cancer patients may change in intensity, nature, and location. Periodic pain assessments are important for all pain patients, but none more than cancer patients. Patients should be encouraged not only to provide numeric or other quantitative ratings of pain intensity, they should also be asked about qualitative pain descriptions. Other symptoms and details about treatments should be considered when evaluating the patient's pain. For example, chemotherapy may induce painful neuropathy that may resolve in whole or in part when chemotherapy concludes (LeQuang and Pergolizzi, 2012).

## Abuse-deterrent formulations

Abuse-deterrent formulations (ADFs) of specific opioid analgesic products are commercially available or in development. These formulations are specifically designed using innovative technologies to resist or deter abuse (Raffa et al., 2012). The goal of an ADF is to make it difficult for a potential abuser to extract the opioid and inject, inhale, or take rectally. ADFs do not prevent oral opioids from being consumed as oral agents. The main types of ADF strategies on the market are tablets that resist crushing, resist dissolving (or turn into a viscous glue), or incorporate an aversive agent such as niacin, or an opioid antagonist (such as naloxone). In theory, an ADF could be created as a prodrug, but no such products are commercially available in development to the best of the authors' knowledge.

In addition to ADFs, abuse-deterrent packaging is being developed, including packaging with radiofrequency identification (RFID). The RFID is a tiny device (chip) which contains essential data in the form of an electronic product code and a serial number unique to each product. RFID technology allows opioids to be tracked throughout the supply chain.

While large-scale longitudinal studies may be required to demonstrate the effectiveness of such ADF products in reducing opioid abuse, when ADF OxyContin was released, the average diversion rate decreased 60% (RADARS System, 2011). Other opioids decreased in diversion when an ADF formulation was introduced to market, but not by such a marked amount. This suggests that ADFs reduce the abuse of that particular product, but possibly not overall opioid abuse. Abusers may simply migrate to a different product.

## Discussion

Not so long ago, a cancer diagnosis usually relegated the patient to palliative care and opioid therapy was viewed as a kindness, with no need for concern about long-term abuse. Today, cancer patients live longer, have extended exposures to opioids, and exhibit at least the same if not greater risk factors for opioid misuse than patients struggling with chronic but nonmalignant pain. A lot has changed since 1988 when the WHO advocated almost the routine use of oral morphine for cancer patients dealing with moderate to severe pain with no particular warnings about long-term exposure (World Health Organization, 1988). Today, clinicians must be mindful that many patients “survive” cancer and live for many years, but sometimes with chronic pain as a consequence of the disease or its treatment.

Cancer pain is prevalent, often severe to very severe, and can markedly diminish the patient's function, quality of life, and even mental health. Pain specialists must provide effective and safe analgesic relief to those dealing with cancer pain, but, it is no longer realistic to dismiss concerns about addiction because the patient has cancer. Indeed, cancer patients may be at special risk for opioid misuse, abuse, and even addiction.

Much has been learned about stratifying risk factors for opioid abuse and managing this risk. Fortunately, most cancer patients will not use opioid analgesics inappropriately, although many will need opioid pain relievers at least for a time period in the trajectory of their illness. Most cancer patients who are prescribed opioid analgesics will derive safe and effective pain relief from these products. Thus, it is neither scientifically sound nor medically compassionate to remove opioid analgesics from the cancer patients who may need them. On the other hand, it is important to recognize that inappropriate opioid use, abuse, and addiction, are possible among cancer patients and steps must be taken to safeguard them. A subset of cancer patients may even be at elevated risk for opioid abuse.

As with much in pain medicine, the care of cancer patients with pain can be summarized in a few practical steps. First, cancer patients with pain must be treated systematically. It is less important what the system is than the fact that a system is in place. This system should include patient interview and a thorough medical history, pain assessments, evaluations, patient education, informed consent, and treatment agreements. These are crucial to success and should not be undertaken haphazardly or casually.

Second, clinicians should educate patients about the risks and benefits of any analgesic therapy and listen carefully to the patient's own goals. Some patients will resist taking opioid analgesics for any number of reasons. Informed consent and a patient agreement offer excellent documentation as well as set forth terms and conditions in unequivocal language. The physician should consider the patient's risk factors for opioid misuse. These risk factors sometimes create a conundrum in that a patient may have a legitimate pain condition necessitating opioid therapy, but clear risk factors for misuse, such as active substance abuse (Savage et al., 2008). Such situations do not necessarily preclude the use of opioid analgesics, but do require close supervision.

Third, it is important to recognize that pain control may rely on multiple simultaneous strategies, including nonpharmacological methods. In some cases, pain may be managed with nonopioids, multimodal nonopioid therapy, a combination of nonopioid, and nonpharmacological methods, or fixed-dose combination products that reduce the amount of opioid use. The use of multiple pain strategies requires careful patient education, but can be very effective, particularly in willing, compliant patients.

Fourth, clinicians should monitor patients closely and discuss the monitoring tools (e.g., pain intensity assessments, UDT, pill counts) in advance. Pain levels must be assessed often. Patients taking opioids should be informed about potential side effects and these effects should be managed proactively. Since opioid-associated side effects can be treatment limiting, it is crucial to long-term therapeutic goals to manage them effectively.

Fifth, clinicians must be vigilant about abuse, even in patients who did not seem to be at risk when opioid therapy commenced. Risk factors change over time (Pergolizzi et al., 2012). When it is suspected or known that a patient is misusing opioids, the physician should be willing to firmly but respectfully refer the patient to a psychologist or addiction specialist. Patients should not be made to feel that they are jeopardizing analgesia by being forthcoming about issues of misuse or addiction, but these issues must be addressed.

## Conclusion

Finally, pain specialists treating pain in cancer patients should be conscientious in documenting their findings, evaluations, and other aspects of care. Cancer patients can be long-term patients with constantly changing needs and this requires diligence in documentation. Best practices should be conscientiously applied to mitigate the potential for abuse, but the clinician and all stakeholders must be aware that as long as opioids are used, there is a degree of risk.

## Author contributions

All authors participated in gathering and interpreting the information; all authors reviewed the final manuscript.

### Conflict of interest statement

The authors declare that the research was conducted in the absence of any commercial or financial relationships that could be construed as a potential conflict of interest.
